# Measuring Metacognition in Cancer: Validation of the Metacognitions Questionnaire 30 (MCQ-30)

**DOI:** 10.1371/journal.pone.0107302

**Published:** 2014-09-12

**Authors:** Sharon A. Cook, Peter Salmon, Graham Dunn, Peter Fisher

**Affiliations:** 1 University of Liverpool, Liverpool, United Kingdom; 2 Royal Liverpool and Broadgreen University Hospitals NHS Trust, Liverpool, United Kingdom; 3 The University of Manchester, Manchester, United Kingdom; Supportive care, Early DIagnosis and Advanced disease (SEDA) research group, United Kingdom

## Abstract

**Objective:**

The Metacognitions Questionnaire 30 assesses metacognitive beliefs and processes which are central to the metacognitive model of emotional disorder. As recent studies have begun to explore the utility of this model for understanding emotional distress after cancer diagnosis, it is important also to assess the validity of the Metacognitions Questionnaire 30 for use in cancer populations.

**Methods:**

229 patients with primary breast or prostate cancer completed the Metacognitions Questionnaire 30 and the Hospital Anxiety and Depression Scale pre-treatment and again 12 months later. The structure and validity of the Metacognitions Questionnaire 30 were assessed using factor analyses and structural equation modelling.

**Results:**

Confirmatory and exploratory factor analyses provided evidence supporting the validity of the previously published 5-factor structure of the Metacognitions Questionnaire 30. Specifically, both pre-treatment and 12 months later, this solution provided the best fit to the data and all items loaded on their expected factors. Structural equation modelling indicated that two dimensions of metacognition (positive and negative beliefs about worry) were significantly associated with anxiety and depression as predicted, providing further evidence of validity.

**Conclusions:**

These findings provide initial evidence that the Metacognitions Questionnaire 30 is a valid measure for use in cancer populations.

## Introduction

Metacognition refers to the knowledge, beliefs and cognitive processes involved in the monitoring, control and appraisal of cognition [1], [2]. The metacognitive model of psychological disorder [2], [3] states that emotional distress is maintained by maladaptive and prolonged patterns of thinking (such as persistent worry or rumination) which are activated and driven by underlying metacognitive beliefs. Two types of metacognitive belief are thought particularly important: positive beliefs about the benefits of specific strategies for coping with distressing thoughts and feelings (e.g. worrying will help me cope); and negative beliefs about the danger and uncontrollability of perseverative thinking (e.g. my worrying is uncontrollable). Positive beliefs about the value of worrying and rumination are thought to activate use of these strategies as a means of regulating emotion and cognition. These strategies become pathological when negative metacognitive beliefs are also activated so that worry or rumination itself becomes the focus of negative appraisal – causing additional worry about worry (meta-worry). In addition, negative beliefs about the need to control thinking may lead to attempts to suppress unwanted thoughts or worries, which typically has a paradoxical effect, increasing their salience and intensifying emotional distress. A second important component of metacognition for understanding emotional distress is the cognitive processes that control and monitor cognition. In particular, the metacognitive model suggests that increased use of selective attention to, and monitoring of, cognition leads to unwanted thoughts and feelings becoming more salient [4]. A recent meta-analysis [5] concluded that Metacognitive Therapy, which challenges metacognitive beliefs, is an effective intervention for both anxiety and depressive disorders. Such findings provide clear support for the value and importance of the metacognitive model for understanding the maintenance of emotional distress. The Metacognitions Questionnaire (MCQ) was developed by Cartwright-Hatton and Wells [6] to explore the metacognitive dimensions that are central in the metacognitive model of emotional disorder. The initial 65-item, questionnaire (MCQ-65) consisted of five subscales based on factor analyses, three of which assess beliefs, including: ‘*Positive beliefs about worry’*; ‘*Negative beliefs about the danger and uncontrollability of worry*’; and ‘*negative beliefs about thoughts in general*’. The remaining two subscales assess the tendency to focus on cognitive events, ‘*Cognitive self-consciousness*’; and confidence in cognitive abilities, particularly memory and attention, ‘*Cognitive confidence*’. The MCQ-65 uses a four-point Likert response scale: 1 (do not agree); 2 (agree slightly); 3 (agree moderately); 4 (agree very much).

However, despite excellent psychometric properties (see Wells [1] for a review), the usefulness of the MCQ-65 was compromised by its length; consequently a shorter 30-item version was developed [7]. This MCQ-30 retained the factor structure and the response scale of the longer measure, with six items selected to represent each metacognitive dimension on the basis of highest factor loading and item clarity in previous studies.

Initial psychometric properties of the MCQ-30 were found, in a sample of 182 student and community participants, to be broadly similar to those of the longer measure [7]. Internal consistency of the subscales ranged from an adequate 0.72 to an excellent 0.93 with adequate test-retest reliability for four out of five subscales (ranging from r = 0.59 ‘*Negative beliefs about worry*’ to r = 0.87 ‘*Cognitive self-consciousness*’). Confirmatory and exploratory factor analysis confirmed an acceptable fit of the original five factor model with most items loading on their predicted factors except in the case of ‘*Need to control thoughts*’ where only three out of six items loaded significantly. In addition, all five subscales were significantly and positively correlated with measures of worry (Penn State Worry Questionnaire, PSWQ [8]) and Trait anxiety (Stait - Trait Anxiety Inventory, STAI [9]) with the subscale ‘*Negative beliefs about worry*’ showing the strongest associations. Further studies have since assessed the psychometric properties of the MCQ-30 in mixed student and community samples in the UK [10] and Turkey [11]. In both cases the original five factor structure was replicated and positive correlations demonstrated with theoretically appropriate measures of worry (PSWQ), anxiety and depression.

Recently, interest has grown in applying the metacognitive model to understanding emotional distress in cancer [12], [13]. Thewes et al [13] used the MCQ-30 to explore for the first time the association of metacognitive beliefs with Fear of Cancer Recurrence (FCR) among young women with early stage breast cancer. They found that the subscale ‘*Negative beliefs about worry*’ was the most highly correlated with FCR and that the MCQ-30 total score accounted for 36% of the variance in this outcome, leading them to conclude that maladaptive metacognitions play an important role in FCR. However, caution is warranted in the interpretation of such findings because without formal psychometric testing we do not yet know how the MCQ-30 operates in a cancer population.

Consequently, the current study aims to explore for the first time the validity of the MCQ-30 in cancer. The primary aim is to explore whether the established 5-factor structure of the MCQ-30 is valid in this population and to investigate the internal consistency of its subscales. A second aim is to explore whether the theoretically expected associations between specific subscales of the MCQ-30 and anxiety and depression demonstrated in previous research (Wells & Cartwright-Hatton, 2004; Spada et al, 2008; Yilmaz et al, 2008) are replicated, thus providing evidence of concurrent validity in this population. As the association of metacognitive beliefs with emotional distress in cancer had not been investigated before this study, this analysis was exploratory with only one *a priori* hypothesis: that the subscale ‘*Negative beliefs about worry*’ would be the main predictor of variance in both anxiety and depression, as this relationship has been consistently documented in mental health [1] physical health [14] and student and community populations [10], [11].

## Methods

### Ethics statement

This research was approved according to UK guidelines, by the NHS North West 5 Research Ethics Committee (reference: 09/H1010/70). There are no conflicts of interest to be declared.

### Participants

Participants were recruited from patients at least 18 years old attending routine pre-treatment clinics at a National Health Service (NHS) teaching hospital, after receiving a diagnosis of primary non-metastatic breast or prostate cancer. Patients were excluded if they had recurrent or metastatic disease, or were considered by the clinical team or researcher to be too distressed or confused to give informed consent.

### Measures

The Metacognitions Questionnaire 30- (MCQ-30) [7] assesses metacognitive beliefs and processes. It comprises five subscales: *‘Positive beliefs about worry’*; *‘Negative beliefs about worry’*; *‘Cognitive confidence’*; *‘Need to control thoughts’*; and *‘Cognitive self-consciousness*. For each subscale, six items are scored 1–4, yielding total scores of 6 to 24. High scores indicate, respectively, more positive and negative beliefs about worry, reduced confidence in memory, greater belief in the need to control thoughts and an increased tendency towards self-focussed attention. The MCQ-30 has excellent internal consistency and good convergent and predictive validity in normal populations [7], [10], [11].

The Hospital Anxiety and Depression scale (HADS) [15] was used to assess anxiety and depression. The HADS is a well-established measure of emotional distress specifically developed for use in physically ill populations. Fourteen items are scored on a 4-point scale yielding two subscale scores of 0-21 with high scores indicating great anxiety or depression. The HADS has been extensively validated for use in cancer [16], [17] and is one of the most widely employed measures of anxiety and depression symptoms in this population.

### Procedure

Data for this study was collected as part of a larger prospective study exploring the association of metacognitive beliefs with emotional distress after cancer [18]. Suitable participants were identified by clinic staff, who gave them recruitment letters and information sheets for the study along with their appointment letters for routine pre-treatment consultations and explained that participation in the research was entirely voluntary. When patients attended the clinic, those willing to see the researcher were given further information and asked for written consent. Participants were asked to complete the study questionnaires in clinic and were given the choice of electronic (hand-held PC) or paper formats. Those unable to complete the questionnaires in clinic took a copies (paper version) home and returned them by post. Twelve months later participants were mailed a second questionnaire pack which they completed and returned by post.

### Data analysis

To explore the validity of the MCQ-30 over time and under different circumstances, the data were analysed separately for both time points (pre-treatment & 12 months later).

Construct validity of the MCQ-30 was first assessed using Confirmatory Factor Analysis (CFA) to test the published five-factor measurement model. As the primary aim of this study was to assess validity rather than achieve the best possible model fit, the decision was taken not to make minor modifications to the model based on the data (unless strongly supported by theory) as such modifications often just reflect idiosyncratic characteristics of the sample [19]. Instead, Exploratory Factor Analysis (EFA) was used to explore whether an alternative model would be more appropriate for this sample. Both sets of analyses (CFA and EFA), were performed in Mplus version 6.12 [20], using the robust weighted least squares estimator (WLSMV [21], [22]) recommended for ordinal categorical data [23]. The EFA tested models up to and including a five-factor structure without dictating where items should load. As previous studies identified MCQ-30 subscales as inter-correlated, an oblique rotation (Geomin) was used to establish the optimum pattern of item loadings. For both analyses (CFA & EFA), adequacy of model fit was assessed based on two incremental fit indices: the Comparative Fit Index (CFI); and the Tucker-Lewis Fit Index (TLI), with values close to 0.95 indicating a well-fitting model [24], and two absolute misfit indices: the Root mean Square Error of Approximation (RMSEA) with values <.05 indicating good fit and 0.5– .08 adequate fit [25]; and the Weighted Root Mean Square Residual (WRMR) with values less than .95 indicating good fit [26]. For the EFA the Standardised Root mean Square (SRMR) was used, instead of the WRMR, with values <.05 indicating good fit. Inter-correlations amongst the five latent factors of the published model were examined and the internal consistency of each subscale assessed using Cronbach's alpha.

Concurrent validity of the MCQ-30 was then assessed (at each time point) by fitting the data to a structural model in which latent variables for anxiety and depression (each indicated by their seven constituent HADS items), were regressed onto the MCQ-30 factors. Adequacy of model fit was again assessed using the fit indices described above. As the MCQ-30 and HADS subscales were not normally distributed and the study sample relatively small, bootstrapping techniques were used to test the robustness of the findings.

## Results

Sample characteristics for the participants at each time point are shown in Table 1.

**Table 1:** Sample characteristics.

	Pre-treatment	12 months follow-up
Total N	229	206
**Age**		
Mean (SD)	61.3 (8.9)	61.5 (9.0)
Range	38–85	39–85
	**n (% of total N)**	**n (% of total N)**
**Gender**		
Female	150 (66)	133
Male	79 (34)	73
**Marital status**		
Married/co-habiting	151 (66)	139
**Live alone**	46 (20%)	37
**Education**		
None	88 (38)	76
School qualifications or higher	132 (58)	121
**Employment**		
Employed (full/part-time)	88 (38)	79
Retired	99 (43)	92
Retired (health)	16 (7)	14
Homemaker	13 (6)	9
Unemployed	10 (4)	9
**Cancer diagnosis**		
Breast	150 (66)	133
Prostate	79 (34)	73
**Tumour grade**		
Low	56 (24)	54
Intermediate	107 (47)	97
High	62 (27)	52

*N.B. Missing data T1 (T2): Marital Status n = 5(5); Live alone n = 3(2); Education n = 9 (9); Employment n = 3(3); Tumour grade n = 4(3).*

### Factorial Structure

Confirmatory factor analysis of the MCQ-30 five factor model showed overall a marginally adequate fit of the model to the data at the pre-treatment assessment: χ^2^ (395)  = 787.448. p<. 01, RMSEA  = .066 (90% CI = .059–.073), CFI  =  .91, TLI  = .90, WRMR  = 1.218.

Exploratory Factor analysis which, unlike CFA, does not dictate where items should load, confirmed that a five-factor solution nevertheless provided the best model. Moreover, the fit indices (χ^2^ (295)  = 439.692. P<.001, RMSEA  = .046 (90% CI = .037–.055), CFI  =  .97, TLI  = .95, SRMR  = 0.046) together indicate a good fit of the model to this data. As shown in Table 2, all items loaded >0.4 on their expected factors [7]. However, as the items were allowed to load freely across any factors, minor discrepancies were observed between the EFA–derived solution and the published five factor model. Specifically, two items, MCQ3 and MCQ13, had their highest loadings on factors other than the expected ones. Item MCQ3 loaded higher on ‘*Negative beliefs about worry*’ (F1) than on its expected factor - ‘*Cognitive self-consciousness*’ (F4). Item MCQ13 had equivalent loadings on both its expected factors - ‘*Need for control over thoughts*’ (F5) - and ‘*Cognitive self-consciousness*’ (F4). Two further items (MCQ5 & MCQ29) also demonstrated significant (>.4) cross-loadings although for both the highest loading remained consistent with the published factor structure.

**Table 2:** Published scale structure and rotated (Geomin) factor loadings from EFA of the Metacognitions Questionnaire-30 at pre-treatment.

MCQ-30 PUBLISHED SCALE STRUCTURE & ITEMS		EFA FACTOR LOADINGS				
		F1	F2	F3	F4	F5
**Subscale:**	**Positive beliefs about worry**					
MCQ-1	Worrying helps me to avoid problems in the future	0.08	**0.66**	0.15	−0.19	0.05
MCQ-7	I need to worry in order to remain organized	0.09	**0.85**	−0.17	−0.03	0.05
MCQ-10	Worrying helps me to get things sorted out in my mind	0.05	**0.88**	−0.05	0.10	−0.14
MCQ-19	Worrying helps me cope	−0.04	**0.85**	0.03	0.12	0.03
MCQ-23	Worrying helps me to solve problems	−0.10	**0.85**	0.05	0.09	0.04
MCQ-28	I need to worry in order to work well	−0.04	**0.79**	0.05	0.10	0.18
**Subscale:**	**Negative beliefs about worry**					
MCQ-2	My worrying is dangerous for me	**0.58**	−0.17	−0.02	0.10	0.14
MCQ-4	I could make myself sick with worrying	**0.65**	−0.01	0.01	0.11	−0.03
MCQ-9	My worrying thoughts persist, no matter how I try to stop them	**0.70**	0.27	0.04	−0.07	−0.03
MCQ-11	I cannot ignore my worrying thoughts	**0.69**	0.39	−0.02	0,01	−0.12
MCQ-15	My worrying could make me go mad	**0.62**	−0.14	0.28	0.20	0.18
MCQ-21	When I start worrying, I cannot stop	**0.76**	0.19	0.08	−0.07	0.09
**Subscale:**	**Cognitive confidence**					
MCQ-8	I have little confidence in my memory for words and names	0.04	0.07	**0.81**	−0.06	−0.03
MCQ-14	My memory can mislead me at times	0.19	−0.03	**0.60**	0.24	−0.07
MCQ-17	I have a poor memory	0.05	0.02	**0.88**	−0.03	−0.05
MCQ-24	I have little confidence in my memory for places	−0.10	0.05	**0.82**	0.04	0.10
MCQ-26	I do not trust my memory	−0.07	−0.03	**0.77**	−0.03	0.30
MCQ-29	I have little confidence in my memory for actions	0.04	0.06	**0.57**	−0.09	0.47
**Subscale:**	**Need for control over thoughts**					
MCQ-6	If I did not control a worrying thought, and then it happened, it would be my fault.	0.39	0.17	−0.14	−0.10	**0.52**
MCQ-13	I should be in control of my thoughts all of the time	−0.04	−0.11	−0.01	**0.41**	**0.41**
MCQ-20	Not being able to control my thoughts is a sign of weakness	0.32	0.06	−0.06	0.03	**0.64**
MCQ-22	I will be punished for not controlling certain thoughts	0.15	0.15	0.21	0.07	**0.72**
MCQ-25	It is bad to thinks certain thoughts	0.13	−0.02	0.11	0.18	**0.50**
MCQ-27	If I could not control my thoughts, I would not be able to function	−0.07	−0.00	−0.05	0.27	**0.64**
**Subscale:**	**Cognitive self-consciousness**					
MCQ-3	I think a lot about my thoughts	**0.56**	0.01	−0.01	**0.43**	−0.06
MCQ-5	I am aware of the way my mind works when I am thinking through a problem	0.14	0.17	−0.07	**0.49**	−0.42
MCQ-12	I monitor my thoughts	−0.03	0.08	−0.12	**0.66**	0.02
MCQ-16	I am constantly aware of my thinking	0.27	0.02	0.05	**0.66**	0.05
MCQ-18	I pay close attention to the way my mind works	−0.02	0.08	0.03	**0.83**	0.02
MCQ-30	I constantly examine my thoughts	0.31	0.05	0.01	**0.55**	0.27

N.B. F1 ‘Negative beliefs about worry’ F2 ‘Positive beliefs about worry’ F3 ‘Cognitive confidence’ F4 ‘Need for control over thoughts’ F5 ‘Cognitive Self-conciousness’; Bold =  loading >.4; Underline  =  highest loading where item loads >.4 on more than one factor.

At the 12 month follow-up, CFA indicated an adequate fit of the data to the published five-factor model: χ^2^ (395)  = 684.184. p<. 01, RMSEA  = .060 (90% CI = .053–.068, (p RMSEA<.05<.015), CFI  =  .95, TLI  = .95, WRMR  = 1.048. Therefore no Exploratory Factor Analysis was performed.

The Mean and SDs of the five MCQ-30 subscales and the correlations amongst the five latent variables (CFA standardised solution) at both time points are presented in Table 3. The internal consistency of the subscales was assessed using Cronbach's alpha (Table 3) and ranged from 0.73 to 0.89 pre-treatment and from .79 to .91 at 12 month follow-up, indicating adequate to excellent internal consistency. At both time points the subscale with the lowest alpha coefficient was ‘*Need for Control*’.

**Table 3:** Descriptive data, internal consistency and inter-correlations among the five latent MCQ-30 factors (CFA standardised solution).

Pre-treatment								
	Mean (SD)	Median (IQR)	POS	NEG	CC	NC	CSC	Alpha
POS	9.2 (3.95)	8 (8.7–9.7)	1	.57**	.29**	.59**	.55**	.89
NEG	11.2 (4.17)	11 (10.6–11.7)		1	.42**	.58**	.64**	.80
CC	10.0 (4.10)	9 (9.5–10.6)			1	.46**	.18*	.85
NC	10.1 (3.68)	9.0 (9.6–10.6)				1	.64**	.73
CSC	13.3 (4.39)	13 (12.7–13.9)					1	.79

12 month follow-up								
	Mean (SD)	Median (IQR)	POS	NEG	CC	NC	CSC	Alpha
POS	9.02 (3.97)	7.0(6–11)	1	.71**	.45**	.68**	.62**	.91
NEG	10.8 (4.48)	10.0 (7–14)		1	.68**	.65**	.73**	.85
CC	11.21 (5.01)	10.0 (7–14)			1	.43**	.37*	.91
NC	9.82 (3.89)	9.0 (7–11)				1	.72**	.79
CSC	12.41 (4.63)	12.0 (8.9–16)					1	.85

N.B. MCQ-30 subscales: ‘Positive beliefs about worry’ (POS); ‘Negative beliefs about worry’ (NEG); ‘Cognitive Confidence’ (CC); ‘Need for control over thoughts’ (NC); ‘Cognitive Self Consciousness’ (CSC) *p<.05; **p<.001.

### Convergent validity

**Figure 1:**
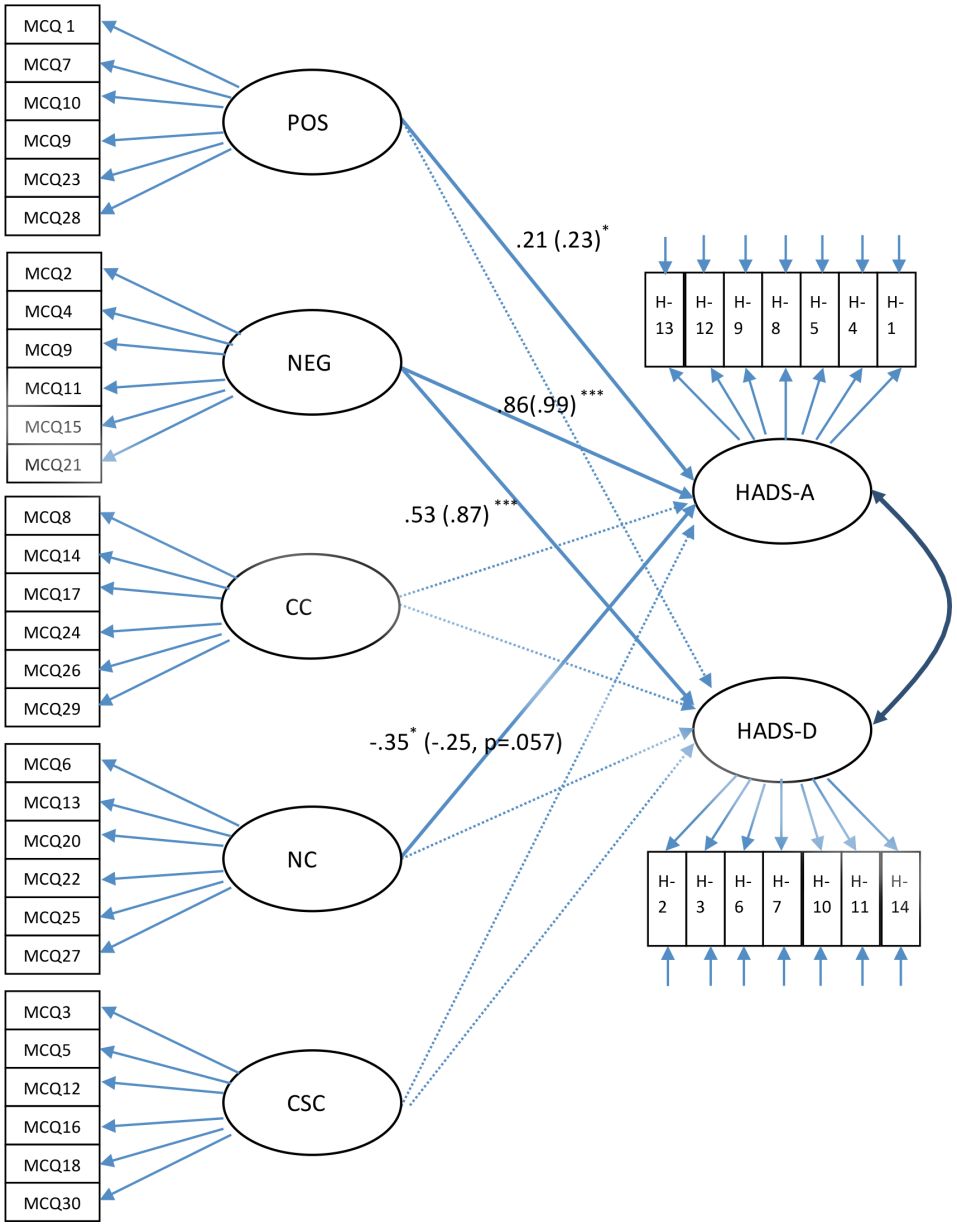
Structural equation model of the relationship between the MCQ-30 and HADS anxiety and HADS depression. N.B. Rectangles indicate observed variables on MCQ-30 (MCQ) or HADS (H); ellipses indicate latent factors. Latent factors: *Positive beliefs about worry* (POS); *Negative beliefs about worry* (NEG); *Cognitive Confidence* (CC); *Need to control thoughts* (NC); *Cognitive Self-consciousness* (CSC); HADS Anxiety (HADS-A); HADS Depression (HADS-D). Figures show standardised path coefficients and their significance at pre-treatment and (in brackets) 12-month follow-up. Errors not shown; ^***^ p<.001 ^**^ p<.01 ^*^ p<.05

The hypothesised model of the relationship between metacognitive beliefs (using the MCQ-30's published factor structure) and concurrent anxiety and depression is shown in Figure 1. Overall, the fit indices for this latent variable SEM (see Table 4) indicated an acceptable fit of the model. At both time points, ‘*Negative beliefs about worry*’ explained significant variance in both anxiety and depression and, as hypothesised, was the strongest of all the predictors. ‘*Positive beliefs about worry*’ also explained variance in anxiety at both time points but not depression. At the pre-treatment time-point *‘Need for control over thoughts’* was associated with fewer symptoms of anxiety, although this association fell just short of significant (p = .057) at the twelve-month follow-up. There was no significant relationship between ‘*Cognitive confidence*’ or *‘Cognitive self-consciousness’* and anxiety or depression at either time-point.

**Table 4:** Fit indices for the pre-treatment and 12-month follow-up SEMs of the relationship between latent factors for the MCQ-30 and HADS anxiety and depression.

Fit Statistics	Pre-treatment	12-month follow-up
**Chi Square Test of Model Fit**		
Value	1354.58	1245.78
Degrees of Freedom	881	881
*p*-value	<.001	<.001
**CFI/TLI**		
CFI	.93	.96
TLI	.93	.95
**RMSEA**		
Estimate (C.I)	.048 (.043–.053)	.045 (.039–.050)
**Weighted Root Mean Square Residual (WRMR)**		
Value	1.147	1.009

## Discussion

The present study provides the first evidence to support the published five-factor structure of the MCQ-30 [7] as valid and replicable in a cancer population. Although at the pre-treatment time point CFA showed only a marginal fit, subsequent EFA confirmed that a five-factor solution still provided the best solution. The improved fit observed for the EFA over the CFA was the result of items being allowed to load freely across any of the factors. However, all items still loaded on their expected factors with only minor discrepancies between the two models. At 12-month follow-up, fit was acceptable and comparable to that reported by the measure's developers [7]. It is not clear why the fit should be slightly better at the 12 month follow-up. The mode of administration differed between the two time points with pre-treatment assessments largely being carried out on hand-held PCs while 12 month follow-up were completed on paper. Improved fit at follow-up might therefore arise because the procedure for this assessment is closer to how the questionnaires have been administered during previous validation studies. Equally, the observed improvement in fit could be partly due to the timing of assessments in that the pre-treatment assessment was conducted relatively soon after diagnosis, during a period that is clinically busy and often emotionally turbulent. In contrast, the twelve month follow-up for most is likely to be a more settled time, at least clinically. However, taken together, these CFA and EFA results suggest that the established five factor structure of the MCQ-30 is valid for use in a cancer population and that it remains valid across one year post-diagnosis and changing illness/treatment circumstances. In addition, the results indicate that the subscales possess good internal consistency comparable to those found in previous studies [7], [10], [11].

Two items (MCQ3 & MCQ13) had their highest loadings on a different factor to that expected. However, only one of these loaded higher on that factor; Item MCQ3 (‘I think a lot about my thoughts’) had its highest loading on ‘*Positive beliefs about worry*’ rather than the expected factor ‘*Cognitive self-consciousness*’. Both of these items have also been found to cross-load on different factors previously [11] although, in that study, item MCQ3 loaded >0.4 on ‘*Negative beliefs about worry*’ not on ‘*Positive beliefs about worry*’ as in the present study.

Preliminary evidence of the measure's convergent validity is provided by the structural equation model of the relationship of the MCQ-30 latent factors with anxiety and depression. As hypothesised, and as shown previously in mental health, physical health, student and community populations, ‘*Negative beliefs about worry*’ was the strongest predictor of both anxiety ([7], [10], [11], [14] and depression [10], [11]. In addition, ‘*Positive beliefs about worry*’ predicted anxiety at both time points. However, in contrast, ‘*Need for control over thoughts*’ was negatively related to anxiety at pre-treatment although this relationship was marginally non-significant at twelve month follow-up. This suggest that participants with lower conviction about the need to control their thinking experience greater anxiety. Such findings are unexpected as previous studies in mental health, student and community samples have indicated that greater belief in the need to control thoughts, predict higher rather than lower levels of anxiety. This result may indicate a difference between this and previously studied mental health, student and community populations. However, further work would be required to establish whether this is a true population difference or just an artefact of the present data.

It is important to note that, by structural equation modelling standards, the study employed a small sample size, which may reduce the stability of the findings. Consequently, further work is required to establish whether the apparent differential item functioning and observed patterns of associations represent real population differences or are idiosyncratic to this data set. In addition, as only breast and prostate cancer patients were included in the study, it remains important to explore whether study findings can be replicated across different cancer diagnoses.

In summary, the current study provides initial evidence that the established five-factor structure of the MCQ-30 is valid for use in a cancer population and that the subscales possess good internal consistency. Positive and negative beliefs about worry were associated with concurrent anxiety and depression as expected, although the negative relationship of anxiety with ‘*Need for control over thoughts*’ is unexpected and therefore intriguing. Despite the limitations discussed above we conclude from this study that, the MCQ-30 is a sufficiently valid measure for assessing metacognitive beliefs and processes in breast or prostate cancer populations in the first year after diagnosis.
